# From Genetics to Epigenetics: New Perspectives in Tourette Syndrome Research

**DOI:** 10.3389/fnins.2016.00277

**Published:** 2016-07-12

**Authors:** Luca Pagliaroli, Borbála Vető, Tamás Arányi, Csaba Barta

**Affiliations:** ^1^Institute of Medical Chemistry, Molecular Biology and Pathobiochemistry, Semmelweis UniversityBudapest, Hungary; ^2^Research Centre for Natural Sciences, Institute of Enzymology, Hungarian Academy of SciencesBudapest, Hungary; ^3^Centre National de la Recherche Scientifique UMR 6214, Institut National de la Santé et de la Recherche Médicale U1083, University of AngersAngers, France

**Keywords:** Tourette Syndrome, genetics, epigenetics, DNA methylation, non-coding RNA, neurological disorders, psychiatric disorders

## Abstract

Gilles de la Tourette Syndrome (TS) is a neurodevelopmental disorder marked by the appearance of multiple involuntary motor and vocal tics. TS presents high comorbidity rates with other disorders such as attention deficit hyperactivity disorder (ADHD) and obsessive compulsive disorder (OCD). TS is highly heritable and has a complex polygenic background. However, environmental factors also play a role in the manifestation of symptoms. Different epigenetic mechanisms may represent the link between these two causalities. Epigenetic regulation has been shown to have an impact in the development of many neuropsychiatric disorders, however very little is known about its effects on Tourette Syndrome. This review provides a summary of the recent findings in genetic background of TS, followed by an overview on different epigenetic mechanisms, such as DNA methylation, histone modifications, and non-coding RNAs in the regulation of gene expression. Epigenetic studies in other neurological and psychiatric disorders are discussed along with the TS-related epigenetic findings available in the literature to date. Moreover, we are proposing that some general epigenetic mechanisms seen in other neuropsychiatric disorders may also play a role in the pathogenesis of TS.

## Introduction

Gilles de la Tourette Syndrome (TS) is a neurodevelopmental disorder characterized by one vocal and multiple motor tics, lasting longer than a year (Robertson, 2000). The prevalence of TS is estimated to be ~1% and it occurs more in males than females, with a ratio of 4 to 1, without differences between social classes. Furthermore, in almost 90% of cases, TS arises along with comorbid neuropsychiatric disorders: in 45–60% with obsessive-compulsive disorder (OCD), while in 60% of cases with attention deficit hyperactivity disorder (ADHD). Anxiety, behavioral disorders, autism spectrum disorders, and learning disabilities are also quite common among individuals with TS (Baron-Cohen et al., 1999; Coffey et al., 2000; Kurlan et al., 2002; Burd et al., 2005; Robertson and Orth, 2006; Cavanna and Termine, 2012; Robertson, 2012).

Tics are defined as sudden movements or vocalizations that are recurrent, rapid, arrhythmic, and stereotyped. They decline in situations of distraction and relaxation, while they increase under stress and anxiety. It is generally preceded by premonitory urges or a sense of inner tension that is reduced or relieved by the performance of the tic (Kwak et al., 2003). Motor tics can be classified as (i) simple, involving single muscle or small group of muscles such as the case of blinking, eye rolling, nose twitching; or (ii) complex, requiring a coordinated pattern of movement or sound like touching, squatting, jumping. In the case of vocal tics, sniffing, throat clearing, snorting, gulping, and coughing are classified as simple; while barking, making of animal noises, and uttering strings of words are classified as complex (Robertson, 2012). The most common manifestation is the eye blinking, which is often the first to appear as well. The typical age of onset ranges from 2 to 21 years with a mean at 7 years. Tourette Syndrome is characterized by a waxing and waning course, peak symptom intensity is usually noted in late childhood. The decline of symptoms is usually in late adolescence or early adulthood (Fusco et al., 2006; Stillman et al., 2009).

## The genetic background of tourette syndrome

Like many other neuropsychiatric disorders, TS also has a complex etiology. Several environmental risk factors have been identified, such as perinatal hypoxia, maternal smoking during pregnancy, exposure to androgens, heat, and fatigue (Swain et al., 2007). These environmental factors may interact with many underlying genetic risk factors. To assess the overall genetic contribution in developing a disorder or trait, heritability estimates can be determined, which usually vary between different studies. Based on a recent meta-analysis of all human twin studies to date, the heritability of tics/tic disorders is 0.45 (Polderman et al., 2015). The two most recent studies on the heritability of TS and tic disorders estimated them to be 0.58 and 0.77, respectively (Davis et al., 2013; Mataix-Cols et al., 2015). The risk for first-degree relatives was significantly higher than for second-degree relatives (odds ratios 18.7 and 4.6, respectively). Despite this relatively large overall genetic contribution, most individual gene variants have only very small effects. Hence, most of the earlier studies attempting to unravel the genetic architecture of TS have been hampered by low statistical power due to small sample sizes and clinical heterogeneity. Linkage and candidate gene association studies have identified a number of chromosomal regions and gene polymorphisms possibly implicated in Tourette Syndrome. The candidates for these small scale studies have traditionally been variations in genes involved in the dopaminergic and serotonergic neurotransmitter systems, generally due to their suspected contribution to the etiology of other psychiatric disorders often comorbid with TS, such as ADHD, OCD, autism, etc.

The studied genetic variations are classified in different categories based on the extent of DNA alteration, including single nucleotide polymorphisms (SNPs), as well as shorter or longer repeat variants, such as variable number of tandem repeats (VNTRs) and copy number variations (CNVs). SNPs are the most common form of genetic variation in humans. A SNP represents the change of a single base-pair (bp) in the individual's DNA sequence. The frequency of a particular SNP can vary from private mutations (i.e., only one individual possesses the variation due to a *de novo* mutation) to very common polymorphisms found in almost half of the population. Millions of SNPs exist in an individual's genome, however, most of them are presumed to be neutral, but some may alter gene expression or cause structural changes in the encoded proteins (and other transcripts). Due to this, many SNPs are known to be associated with various human traits and diseases. VNTRs are polymorphisms in the genome where a short sequence of nucleotides (usually up to 100 bp) is organized in multiple copies, which are clustered together and oriented in the same direction. The copy number can vary between individuals and this may influence gene expression or protein structure. CNVs represent much larger genetic rearrangements (usually thousands to millions of base-pairs in length) and therefore change the copy number of genes within the region due to the deletion or duplication of a chromosomal segment. Gene variants implicated in TS are summarized in Tables 1, 2.

**Table 1:** **Multiple (positive) findings**.

**Gene**	**Full gene name**	**PUBMED ID**	**Type of analysis**	**Variation**	**Sample size**	**Ethnicity**	**References**
AADAC	Arylacetamide deacetylase	13	CNV, meta-analysis	Deletion	243 TS patients, 1571 controls	European	Bertelsen et al., 2016
BTBD9	BTB domain containing 9	114781	SNP	rs9296249	110 TS patients, 440 controls	Han Chinese	Guo et al., 2012
			SNP	rs4714156, rs9296249, rs9357271	322 TS patients, 290 controls	French Canadian	Rivière et al., 2009
DRD2/ANKK1	Dopamine receptor D2/ankyrin repeat and kinase domain containing 1	1813/255239	SNP	Taq I A/rs1800497	523 TS patients, 564 controls	European, Asian	Yuan et al., 2015
			SNP	rs6279, rs1079597, rs4648318	69 TS trios	Antioquian	Herzberg et al., 2010
			SNP	Taq I A/rs1800497	151 TS patients, 183 controls	Taiwanese	Lee et al., 2005
			SNP	Taq I A/rs1800497	274 TS patients, 714 controls	European	Comings et al., 1996b
			SNP	Taq I A/rs1800497	147 TS patients, 314 controls	European	Comings et al., 1991
DRD4	Dopamine receptor D4	1815	VNTR	48 bp exon 3 VNTR	291 TS patients (218 trios), 405 controls	Han Chinese	Liu et al., 2014
			VNTR	48 bp exon 3 VNTR	110 TS trios	French Canadian	Díaz-Anzaldúa et al., 2004
			VNTR	48 bp exon 3 VNTR	64 TS family trios	European	Grice et al., 1996
HDC	Histidine decarboxylase	3067	SNP	rs854150, rs1894236	520 TS families	European	Karagiannidis et al., 2013
			SNP	rare coding mutation (W317X)	720 TS patients, 360 controls	NA	Ercan-Sencicek et al., 2010
IMMP2L	IMP2 inner mitochondrial membrane peptidase-like (*S. cerevisiae*)	83943	CNV	Chromosomal deletion	188 TS patients, 316 controls	European (Danish)	Bertelsen et al., 2014
			CNV	Chromosomal translocation	1TS patient	European (British)	Patel et al., 2011
			SNP	rs112636940	258 TS trios	French Canadian	Díaz-Anzaldúa et al., 2004
			CNV	Chromosomal duplication	1 TS patient		Kroisel et al., 2001
			CNV	Chromosomal duplication	1 TS patient	NA	Petek et al., 2001
			CNV	Chromosomal translocation	1 TS patient	NA	Boghosian-Sell et al., 1996
MAOA	Monoamine oxidase A	4128	VNTR	Promoter	110 TS trios	French Canadian	Díaz-Anzaldúa et al., 2004
			VNTR	Exon	375 TS patients, 280 controls	European	Gade et al., 1998
NRXN1	Neurexin 1	9378	CNV	Chromosomal deletion	210 TS patients	Latin American	Nag et al., 2013
			CNV	Chromosomal deletion	111 TS patients, 73 controls		Sundaram et al., 2010
SLC6A3 (DAT1)	Solute carrier family 6 (neurotransmitter transporter), member 3	6531	VNTR	40 bp VNTR	103 TS trios	European (Hungarian)	Tarnok et al., 2007
			SNP	rs6347	266 TS patients, 236 controls	European	Yoon et al., 2007
			VNTR	40 bp VNTR	110 TS trios	French Canadian	Díaz-Anzaldúa et al., 2004
SLITRK1	SLIT and NTRK like family member 1	114798	SNP	rs9546538, rs9531520, rs9593835	NA	Japanese	Inai et al., 2015
			SNP	rs9593835, r9546538	375 TS families	European	Karagiannidis et al., 2012
			SNP, CNV	var321, chromosomal inversion,	174 TS patients, 2148 controls	European	O'Roak et al., 2010
			SNP	rs9593835	154 TS families	Canadian	Miranda et al., 2009
			SNP	var321	174 TS patients	European (Caucasian)	Abelson et al., 2005

**Table 2:** **Single (positive) findings**.

**Gene**	**Full gene name**	**PUBMED ID**	**Type of analysis**	**Variation**	**Sample size**	**Ethnicity**	**Reference**
ADORA1	Adenosine A1 receptor	134	SNP	rs2228079	162 TS patients, 210 controls	European (Polish)	Janik et al., 2015
ADORA2A	Adenosine A2a receptor	135	SNP	rs5751876	162 TS patients, 210 controls	European (Polish)	Janik et al., 2015
BDNF	Brain-derived neurotrophic factor	627	SNP	rs6265	331 TS patients, 519 controls	Han Chinese	Liu et al., 2015a
CHRNA7	Cholinergic receptor, nicotinic, alpha 7 (neuronal)	1139	CNV	Chromosomal duplication	1 TS family	European (Danish)	Melchior et al., 2013
CNTNAP2	Contactin associated protein-like 2	26047	CNV	Chromosomal insertion/translocation	1 TS family	NA	Verkerk et al., 2003
COL8A1	Collagen, type VIII, alpha 1	1295	CNV	Chromosomal duplication	210 TS patients	Latin American	Nag et al., 2013
COMT	Catechol-O-methyltransferase	1312	CNV	Chromosomal duplication	1 TS patient	NA	Clarke et al., 2009
DLGAP3	Discs large homolog associated protein 3	28512	SNP	rs11264126	289 TS trios	NA	Crane et al., 2011
DPP6	Dipeptidyl-peptidase 6	1804	CNV	Chromosomal deletion	1 TS family	European (Italian)	Prontera et al., 2014
DRD3	Dopamine receptor D3	1814	SNP	Msc I polymorphism	139 TS patient, 91 controls	European	Comings et al., 1993
GDNF	Glial cell derived neurotrophic factor	2668	SNP	rs3096140	201 TS patients, 253 controls	American	Huertas-Fernández et al., 2015
GRIN2B	Glutamate receptor, ionotropic, N-methyl D-aspartate 2B	14812	SNP	rs1805476, rs1805502	261 TS nuclear families	Han Chinese	Che et al., 2015
GSTP1	Glutathione S-transferase pi 1	2950	SNP	rs6591256	121 TS patients, 105 controls	Taiwanese	Shen et al., 2014
HTR2C	5-hydroxytryptamine receptor 2C	3358	SNP	rs3813929, rs518147	87 TS patients, 311 controls	European	Dehning et al., 2010
IL1RN	Interleukin 1 receptor antagonist	3557	SNP	IL1B/IL1RN	159 TS patients, 175 controls	Taiwanese	Chou et al., 2010
LHX6	LIM homeobox 6	26468	SNP	rs3808901	222 TS trios	European	Paschou et al., 2012
NLGN4	Neuroligin 4, X-linked	57502	CNV	Chromosomal deletion	1 TS family	Irish-English	Lawson-Yuen et al., 2008
NTN4	Netrin 4	59277	SNP, meta-analysis	rs2060546	1008 TS patients, 1220 controls	European/French Canadian	Paschou et al., 2014
OLFM1	Olfactomedin 1	10439	CNV	Chromosomal translocation	176 TS patients	European (Danish)	Bertelsen et al., 2015
PARP1	Poly (ADP-ribose) polymerase 1	142	SNP	rs1805404	123 TS patients, 105 controls	Taiwanese	Wu et al., 2013a
RUNX1T1 (CBFA2T1)	Runt related transcription factor 1; translocated to, 1 (cyclin D related)	862	CNV	Chromosomal translocation	1 TS family	NA	Matsumoto et al., 2000
SLC6A4 (SERT)	Solute carrier family 6 member 4	6532	SNP	rs25531, rs25532	151 TS patients, 858 controls	European	Moya et al., 2013
TBCD	Tubulin folding cofactor D	6904	SNP	rs662669, rs3744161	4 TS families 105/357, 96 TS families	European	Paschou et al., 2004
TDO2	Tryptophan 2,3-dioxygenase	6999	SNP	Intron 6, G/T variant	NA	NA	Comings et al., 1996a
TDP1	Tyrosyl-DNA phosphodiesterase 1	55775	SNP	rs28365054	122 TS patients, 105 controls	Taiwanese	Wu et al., 2013b
TNF	Tumor necrosis factor	7124	SNP	rs1800629	117 TS patients, 405 controls	European	Keszler et al., 2014
TPH2	Tryptophan hydroxylase 2	121278	SNP	rs4565946	98 TS patients, 178 controls	European (German)	Mössner et al., 2007
XRCC1	X-ray repair complementing defective repair in Chinese hamster cells 1	7515	SNP	rs25487	73 TS patients, 158 controls	Han Chinese	Lin et al., 2012

Most genetic findings implicated in the pathogenesis of Tourette Syndrome to date are inconsistent and studies yielding positive results lack replication in other independent cohorts. On the other hand, some nominally significant candidate gene associations might suffer from lack of proper statistical correction for multiple testing (e.g., Bonferroni correction). However, there are some examples for positive replications as well, these are summarized in Table 1. During the recent years meta-analyses confirmed the role of SLITRK1, NTN4, DRD2, DRD4 and AADAC gene polymorphisms in TS (Liu et al., 2014; Paschou et al., 2014; Yuan et al., 2015; Bertelsen et al., 2016). Here, we will review most of the genes that have been consistently replicated in TS. Single positive findings are summarized in Table 2 and are not discussed in detail.

SLITRK1 is a member of the SLIT and TRK family, a type-I transmembrane protein with an extracellular leucine-rich repeat domain homologous to SLIT (Aruga and Mikoshiba, 2003). It is involved in the control of neurite outgrowth and it is expressed during both embryonic and postnatal development of the cortex, thalamus and the basal ganglia, the neuroanatomical structures believed to be affected in TS (Proenca et al., 2011). The gene coding for SLITRK1 is one of the most studied in relation with Tourette Syndrome. In 2005, a de novo inversion at chromosome 13q33.1 and two additional rare variants were identified in TS patients in the region including a single nucleotide deletion causing a frameshift and a truncated protein and a mutation in the 3′ untranslated region (3′UTR) at the putative binding site for microRNA hsa-miR-189 (Abelson et al., 2005). Since the original discovery several studies in various populations have attempted to replicate the association with controversial results. In a family study of Canadians, a common variation rs9593835 and two haplotypes were found to be associated with the disorder (Miranda et al., 2009). These results were also confirmed in a large sample of European trios with TS (Karagiannidis et al., 2012). A recent study on SLITRK1 found a significant difference in the distribution of haplotypes consisting of SNPs rs9546538, rs9531520, and rs9593835 between Japanese patients and controls (Inai et al., 2015).

Only one genome-wide association study (GWAS) has been published to date in TS by a large international consortium including a sample of 1285 cases and 4964 ancestry-matched controls (Scharf et al., 2013). After correction for multiple testing, none of the half a million studied SNPs reached genome wide significance (*p* < 5^*^10^−8^), however top hits were enriched for gene variants expressed in the brain and some of them coincide with previous candidate genes. The top hit was rs7868992 (*p* = 1.85^*^10^−6^), which is located in an intronic region of the *COL27A1* gene (collagen, type XXVII, alpha 1). COL27A1 is a fibrillar collagen primarily expressed in cartilage, but it is also expressed in the cerebellum during several stages of human development (Pace et al., 2003), however, its function in the developing nervous system is unknown (Fox, 2008). Another study in 260 Chinese trios assessed the preferential transmission of the rs7868992 and two other COL27A1 gene variants (rs4979357 and rs7868992) by transmission disequilibrium test (TDT) and found the latter two variants nominally significant, however these results did not survive correction for multiple testing (Liu et al., 2015b).

A replication study with the top 42 SNPs of the original GWAS was performed with a sample of over 600 cases and 600 controls and the meta-analysis yielded a top signal at rs2060546 with *p* = 5.8^*^10^−7^ in proximity of the NTN4 gene on chromosome 12q22, which codes for netrin 4, an axon guidance protein expressed in the developing striatum. Many of the previous findings (26 out of 42) showed a similar trend underlining the reliability of the GWAS hits as true risk factors for TS (Paschou et al., 2014).

Dopamine receptors, especially DRD2 and DRD4 are two of the most widely studied candidate genes in the field of psychiatric genetics. The dopamine D2 receptor (DRD2) located on chromosome 11p23.2 was characterized previously with TaqIA, TaqIB and TaqID SNPs based on restriction digestion of these polymorphic sites with the TaqI restriction endonuclease enzyme (Vereczkei et al., 2013). Later it turned out that the TaqIA cleavage site is located ~10 kilobases downstream from the DRD2 gene, in exon 8 of the ANKK1 (ankyrin repeat and kinase domain containing 1) gene (Neville et al., 2004), which is a member of the serine/threonine kinase family. The TaqIA polymorphism (rs1800497) causes an amino acid change in ANKK1 (Glu713Lys), which seems to have a significant effect on the specificity of substrate binding. The protein product of the ANKK1 gene was considered as a negative regulator of the transcription factor NF-kB (Nuclear Factor-Kappa B) (Meylan and Tschopp, 2005). Moreover, the expression levels of NF-kB-regulated genes were shown to be altered by the TaqIA variant in an *in vitro* luciferase system (Huang et al., 2009). Since DRD2 is regulated by NF-kB (Fiorentini et al., 2002; Bontempi et al., 2007) it could be assumed that this ANKK1 variant can indirectly affect DRD2 receptor density. It is also possible, however, that the TaqIA SNP is only a marker of other functional DRD2 variants associated with a number of psychiatric disorders, such as the strongly linked TaqIB (Ponce et al., 2009).

A recent meta-analysis on the association of the TaqIA SNP rs1800497 and TS based on a number of previous case-control studies (Table 1) comprising a sample of over 500 cases and 500 controls, as well as TS trios concluded that this variant is indeed a risk factor for the development of the disorder (Yuan et al., 2015).

Another widely studied polymorphic dopamine receptor is thedopamine D4 receptor gene (DRD4) The DRD4 gene located on chromosome 11p15.5 is highly polymorphic, containing over 200 SNPs and several VNTRs. A 48 bp long VNTR ranging from 2 to 11 repeats in exon 3 of the gene changes the length of the third intracellular loop of the receptor (Vereczkei et al., 2013) with a possible effect on downstream signaling efficiency by inhibiting the adenylyl cyclase enzyme (Van Tol et al., 1992). The DRD4 7 repeat allele seems to show decreased sensitivity to dopamine compared to the 4 repeat allele (Asghari et al., 2002) and according to more recent neurobiological findings it does not form heteromers with D2 receptors in the striatum (Borroto-Escuela et al., 2011). The D4 receptor is mainly expressed in cortical and limbic regions in the CNS and carriers of the 7 repeat allele were shown to have higher susceptibility to various addictive behaviors, ADHD, as well as several other psychiatric traits.

A number of candidate gene studies have addressed the issue of possible relevance of the exon 3 VNTR in DRD4 in TS, and a couple of small family studies have found a positive association (Table 1). A combined family and case-control study in a Han Chinese TS population revealed significant transmission disequilibrium for the 2 repeat and the 7 repeat alleles. The results suggest that the 2 repeat allele might play a protective role, while the common 4 repeat may predispose to TS (Liu et al., 2014).

Arylacetamide deacetylase (AADAC) is an enzyme involved in neutral lipid lipolysis, detoxification, and drug metabolism and is mainly expressed in the liver (Quiroga and Lehner, 2011). However, its expression in different brain regions has also been confirmed with a so far unknown physiological relevance. A recent CNV-analysis in a smaller Danish TS cohort followed by a meta-analysis of a large European sample of over 1000 TS patients and 100,000 controls confirmed the role of AADAC deletions in TS pathogenesis (Bertelsen et al., 2016).

The IMMP2L gene located on chromosome 7q31 encodes a protein involved in processing the signal peptide sequences used to target mitochondrial proteins into the mitochondrium. Its association with TS was first discovered in a family with a balanced 7;18 translocation (Boghosian-Sell et al., 1996). A recent CNV study in a Danish cohort of 188 TS patients reported a 5′-end intragenic deletion of IMMP2L in seven of these patients (Bertelsen et al., 2014). Interestingly, in 4 of the 7 cases, the deletion was within intron 3. The frequency of this IMMP2L deletion was significantly higher than that of the control population. Notably, IMMP2L has been implicated in other neuropsychiatric disorders, such as autism and ADHD (International Molecular Genetic Study of Autism Consortium, 1998; Elia et al., 2010).

Histidine Decarboxylase (*HDC*), located on chromosome 15q21.2, encodes a member of the group II decarboxylase family that converts the amino acid L-histidine into histamine, a biogenic amine that can act as a local mediator released from mast cells during an immune reaction, as well as a monoaminergic neurotransmitter in the CNS. A premature termination codon (W317X) in the HDC gene was discovered in a large multigenerational family, where the father and all eight of his children were affected with TS had the non-sense variant (Ercan-Sencicek et al., 2010). On the other hand, a study of 100 Han Chinese TS patients failed to confirm the association of this non-sense mutation in HDC with the disorder (Lei et al., 2012). However, a subsequent large family study of 520 European trios with Tourette Syndrome transmission disequilibrium test (TDT) found SNPs rs854150 and rs1894236 to be over-transmitted in patients, confirming the role of HDC in the development of TS (Karagiannidis et al., 2013). Interestingly, HDC knockout mice have also been proposed as a genetic animal model of TS. The KO animals exhibited potentiated tic-like stereotypies, recapitulating a core phenomenology of the disorder (Castellan Baldan et al., 2014; Xu et al., 2015).

The NRXN1 gene located on chromosome 2p16.3 codes for an important mediator of cell-cell interactions in the central nervous system and it has been implicated in neuropsychiatric disorders, such as autism and schizophrenia (Vrijenhoek et al., 2008; Glessner et al., 2009). A rearrangement of ~400 Kb within exons 1-3 of NRXN1 was recently found in TS patients (Nag et al., 2013). Notably, this result is also consistent with a previous analysis reporting the presence of a CNV comprising NRXN1 in a Danish TS cohort (Sundaram et al., 2010).

For a more detailed review on the genetic background of TS and the description of candidate genes where no positive replications were published in the last few years, such as BTBD9, SLC6A3 (DAT1) and MAO-A, see recent review papers by (Paschou, 2013; Pauls et al., 2014).

## From genetics to epigenetics

As described in the previous section, etiology of Tourette Syndrome has a considerable genetic component. However, it is evident that environment also plays an important role in TS, since discordance between monozygotic twins is not rare, while both prenatal (smoke, alcohol abuse, low birth weight, etc), and perinatal (complicated birth) risk factors were reported. Furthermore, it has also been hypothesized that TS might arise as a consequence of autoimmune mechanisms following Group A β-hemolytic streptococcal infection (GABHS) (Swedo et al., 1998; Snider and Swedo, 2003), a condition labeled as pediatric autoimmune neuropsychiatric disorder associated with streptococcal infection (PANDAS). Finally, the course of the disease is also influenced by environmental factors.

As seen earlier, based on family and twin studies the heritability of TS lies approximately between 50 and 80%, which is a figure often seen in case of other psychiatric disorders. However, gene polymorphisms reported by association studies of these disorders rarely account for more than just a fraction of the overall estimated genetic variance. This phenomenon of “missing heritability” may, in part, be explained by various epigenetic mechanisms arising from the dynamic interaction between the environment and an individual's genome.

**Figure 1:**
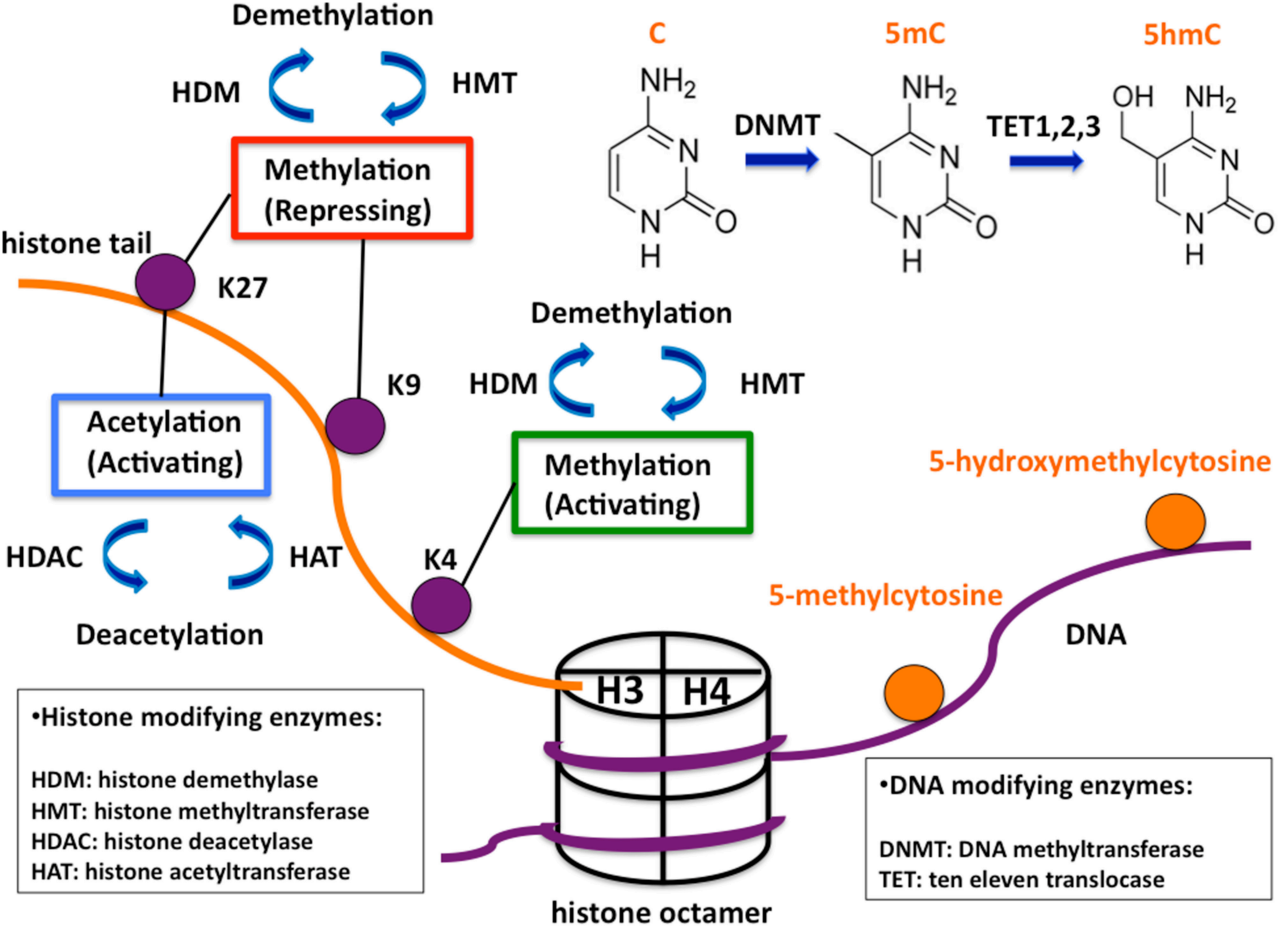
**Overview of common covalent epigenetic modifications**. A schematic nucleosome and examples of potential epigenetic modifications are shown. The histone octamer is represented in a cylindrical form with one pair of histones H3/H4 indicated. The protruding H3 histone tail and DNA are indicated in orange and purple, respectively. Oppositely, histone and DNA modifications are shown in purple and orange. The functional roles of histone modifications are indicated in colored boxes. The enzyme families catalyzing the modifications are listed in boxes below. The enzymatic links between the different cytosine modifications are shown in the upper right corner of the figure.

Cells are reacting to acute and chronic environmental changes by altering their gene expression state, which can be considered as their adaptive reaction. The first step in this adaptive reaction is the modification of the chromatin structure. Chromatin is the macromolecular complex containing DNA and nuclear proteins/histones. The nucleosome is the building unit of chromatin. It consists of two of each of the four core histones (H3, H4, H2A, and H2B) forming an octamer wrapped around twice by DNA (Figure 1). The positively charged N-terminal histone tails are prone to undergo several modifications (acetylation, methylation, phosphorylation, etc.). Similarly, DNA can be methylated. These covalent modifications influence the chromatin structure and lead to changes of the transcriptional state of genes. Even though these changes are dynamic, they can be maintained through cell divisions and from one generation to the next. This mechanism, which does not involve the DNA sequence, but allows the transmission of acquired traits through mitosis and sometimes through a few generations, is called epigenetic inheritance. Another main but chromatin-independent epigenetic mechanism is via non-coding RNAs, which also play a crucial role in gene expression regulation often together with chromatin-related mechanisms.

## Epigenetic modifications

### DNA methylation

The most common epigenetic modification of DNA is the covalent attachment of a methyl group to the C5 position of cytosines. In mammals methylation occurs almost exclusively in CpG dinucleotides (Ziller et al., 2011). Most of the CpG dinucleotides of the genome are present in regions with low GC abundance (Bird, 1993). These CG dinucleotides are generally methylated in all cell types. Methylation leads to high mutation rate, since the frequent oxidative deamination of 5-methyl-cytosine (5 mC) results in thymine (Antonarakis et al., 2000; Baba et al., 2011). This mutation is inefficiently repaired and thus its transmission rate is high. Therefore, low CpG frequency (10% of the expected) characterizes the non-coding part of the genome and CpG cytosines progressively disappear from these genomic regions. However, short CpG-rich sequences (CpG islands – CGI) are frequently found in gene regulatory regions (Bird et al., 1985; Deaton and Bird, 2011). CGIs often show tissue-specific methylation pattern and are frequently unmethylated (only) in germ cells (Smallwood et al., 2011; Zeng et al., 2014). Thus, the important gene regulatory sequences are preserved from a high mutation rate maintaining their biological function.

DNA methylation is tightly linked to gene expression. The more a regulatory region is methylated the less the gene is expressed. Several gene expression regulating DNA binding proteins (e.g., transcription factors) are sensitive to the methylation of their target sequence. Some of them cannot bind if the sequence is methylated (e.g., CTCF, E2F family, Myc, CREB; Hark et al., 2000; Blattler and Farnham, 2013), while others require methylated DNA for binding (e.g., MeCP2 methyl-CpG-binding protein 2 or Kaiso, a zinc finger protein; Lewis et al., 1992; Prokhortchouk et al., 2001; Arányi et al., 2005; Smith and Meissner, 2013). By the stabilization of different chromatin states, DNA methylation contributes to cell differentiation, cellular memory, X chromosome inactivation, and other nuclear processes. Although initially it was considered to be a static epigenetic mark, DNA methylation dynamically changes (Kangaspeska et al., 2008; Métivier et al., 2008; Yamagata et al., 2012b) and different enzymes ensure the equilibrium state.

DNA methylation is catalyzed by DNA methyl-transferase (DNMT) enzymes, which either establish or maintain the methylation pattern. DNMT1 is a maintenance methyl-transferase and it preferentially methylates DNA methylated only on one strand in order to preserve and reestablish the pattern of methylation after DNA replication (Pradhan et al., 1999; Mohan and Chaillet, 2013). By doing so, during DNA replication DNMT1 is enriched at the replication fork and reproduces the methylation pattern based on the original template strand (Schermelleh et al., 2007). DNMT3A and DNMT3B are *de novo* methyl-transferases capable of establishing new patterns of methylation. DNA demethylation is mainly performed by members of the ten-eleven-transferase (TET) enzyme family through the hydroxylation of the methyl group. This leads to the formation of 5-hydroxymethyl-cytosine (5 hmC), which is lost after further oxidation by the same enzyme (Hashimoto et al., 2015). While 5 mC nucleotides represent a few percent (typically between 3 and 7%) of all genomic cytosines in cells and cell lines, 5 hmC is much less abundant constituting only 0.01–1% of all cytosines (Globisch et al., 2010). Interestingly, 5 hmC is rather frequent in primary cells and particularly in the brain (Globisch et al., 2010). Different data also indicate that 5 hmC does not have a general transcriptional repression effect as 5 mC does (Wu et al., 2011; Kang et al., 2015; Li et al., 2016).

During development, genome-wide methylation changes occur before the differentiating cells acquire the adult-type methylation profiles. Similarly, induction of pluripotent stem cells or differentiation of stem cells in their physiological niches is accompanied by general DNA methylation changes. It is a dynamic and lifelong feature of DNA methylation. Local methylation changes happen in response to environmental factors, such as hormonal and metabolic effects or even early childhood stress (see later). The affected genes are silenced for long periods potentially through several generations. Toxic molecules, infections and hereditary or acquired diseases can have similar effects (Yamagata et al., 2012a). However, these alterations can undergo rapid reversion under appropriate environmental conditions. Due to the different environmental factors acting on each individual separately, DNA methylation changes throughout aging generate increasing methylation pattern differences between monozygotic twins, which leads to progressively appearing phenotypical variability (Fraga et al., 2005).

### Histone modifications

Histones are small, globular proteins, which are highly conserved in all eukaryotes. As mentioned earlier, the core histones building up the nucleosomes have N-terminal protruding tails, which are particularly prone to undergo posttranslational modifications (Allfrey et al., 1964). These epigenetic modifications include acetylation, methylation and phosphorylation. They play an important role in different nuclear processes, such as replication, DNA repair, transcription, and chromatin structure stabilization (Kouzarides, 2007; Bannister and Kouzarides, 2011). Although it was reported several decades ago that histones might undergo covalent modifications, their intensive investigation started only in the late 1990s.

The initial studies identified the lysine residues as targets of acetylation and revealed that they are essentially located on the H3 and H4 histone N-terminal tails. These modifications have rapid turnover (Zee et al., 2010). The reactions are catalysed by a high number of histone acetyl-transferases (HAT) and histone deacetylases (HDAC and Sirt) (Kuo and Allis, 1998; Legube and Trouche, 2003). Some complexes with HAT activity (e.g., p300 and CBP–CREB-binding protein) recruit also transcription factors and RNA PolII (Sakamoto et al., 2004). Thus, not surprisingly, lysine acetylation marks transcriptionally active euchromatic gene regulatory regions. For example, H3K27 (lysine 27 of histone H3) acetylation identifies active regulatory elements and separates active and inactive enhancers (ENCODE Project Consortium, 2012). H4 acetylation also indicates active chromatin territories; the acetylation of the two histones often occurs simultaneously. Acetylation profiles are inherited during DNA replication. The molecular mechanisms are still unclear and there might be several. According to one of them, histones with their epigenetic modifications are randomly distributed between the two new DNA molecules while the new chromatin is forming. Than newly synthesized histones are deposited and they rapidly acquire similar modifications to the old ones (Budhavarapu et al., 2013).

After understanding the role of histone acetylation, studies on histone methylation begun. Histone methylation has a much more complex pattern than acetylation since both arginines and lysines can be modified. Furthermore, arginines can be mono- or di-methylated, while lysines can be mono-, di-, or tri-methylated (Bannister and Kouzarides, 2011; Jahan and Davie, 2015; Greenblatt et al., 2016). Dozens of enzymes catalyse these post-translational modifications and their removal. The different enzymes are very selective and are capable to catalyse only one or two specific reactions (such as mono- and di-methylation of a specific lysine but not tri-methylation). Different histone methylations are specifically associated with various gene or chromatin regions. For example, H3K4me3 (trimethylation of lysine 4 of histone H3) marks transcription start sites (TSS) and it is characteristic of active promoters in the euchromatin (Santos-Rosa and Caldas, 2005). In addition, H3K36me3 is associated with actively transcribed gene bodies (Edmunds et al., 2008), while H3K27me3 typically occurs in transcriptionally repressed, heterochromatic regions (Bracken et al., 2006).

### Networks of epigenetic modifications

In the different chromatin regions, complex patterns of histone and DNA modifications co-occur, which led to the formulation of the “histone code” hypothesis (Jenuwein and Allis, 2001). According to this hypothesis distinct patterns of chromatin modifications at any given genomic region would have the same meaning. These patterns are read by specific proteins, which then execute their local roles accordingly. However, it turned out that the histone code is most probably highly redundant.

Still, the epigenetic modifications are recognized by chromatin binding proteins. These proteins then often antagonize or promote the removal or catalysis of other covalent marks leading to the formation of complex patterns (Bannister and Kouzarides, 2011). Other proteins sensitive to the epigenetic pattern play important role in nuclear processes (e.g., γH2AX, a phosphorylated histone, participating in DNA repair). The proteins sensing and modifying the epigenetic marks are called readers, writers or erasers. Certainly, these proteins bind the chromatin with different affinity and therefore, they reside there for a shorter or longer time period depending on the local environmental context. The networks of modifications are therefore stochastically self-assembling and disassembling with various probability depending on the environmental conditions (Jeltsch and Jurkowska, 2014). This rapidly changing feature of the network makes the system particularly efficient in reacting to environmental stress (e.g., starvation, oxidative stress, or viral infection (Yamagata et al., 2012a) and modulating gene expression states in order to maintain the cellular homeostasis.

**Figure 2:**
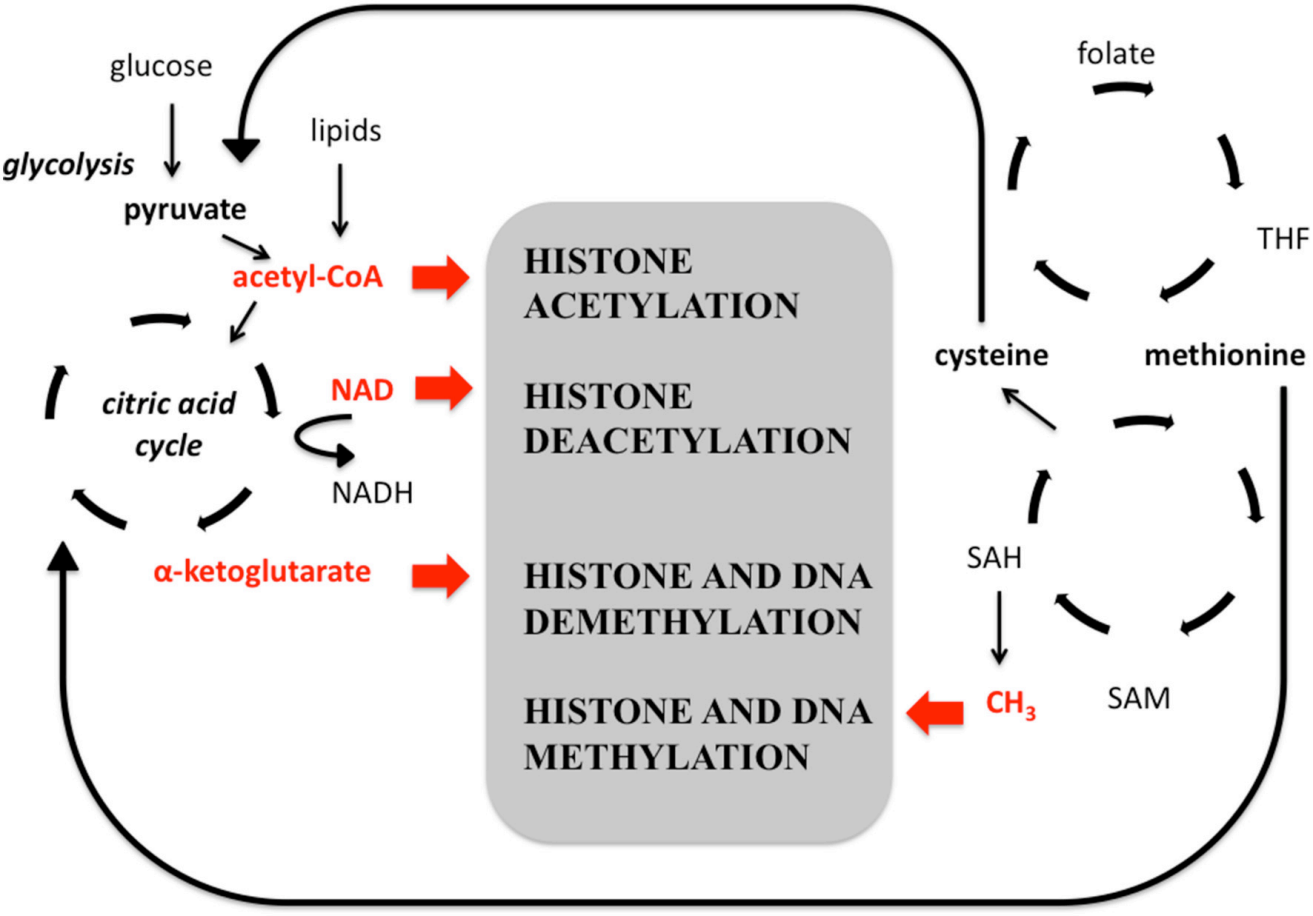
**The relationship between epigenetic modifications and intermediary metabolism**. Glycolysis, lipid metabolism, citric acid cycle, amino acid metabolism, and folate/SAM cycles are tightly linked to epigenetic modifications (shown in the middle), since their products and cofactors (shown in red) are substrates of enzymes catalyzing the epigenetic modifications. Acetyl-coenzyme A and NAD contribute to histone acetylation and deacetylation, respectively. Methyl groups and alfa-ketoglutarate participate in the methylation and demethylation of both histones and DNA. NAD: nicotinamide adenine dinucleotide, THF: tetrahydrofolate, SAH: S-adenosylhomocysteine, SAM: S-adenosylmethionine.

Therefore, it is not surprising that enzymes catalysing the addition or removal of covalent post-translational modifications of histones and DNA are closely linked to intermediary metabolism (Figure 2). To acetylate lysine residues, HAT enzymes use acetyl-CoA, a key molecule in carbohydrate and fat metabolism. Class III histone deacetylases (Sirt) need NAD+ for their activity (Vaquero et al., 2007). High level of energy intake leads to hyperacetylated histones, while low energy intake favors histone hypoacetylation. Methylation of histones and DNA needs S-adenosyl-methionin (SAM) as a cofactor, the methyl donor in biochemical reactions. The reaction also needs folate to regenerate SAM. The TeT (Ten-eleven Translocase) enzymes are oxygenases, which catalyse the demethylation reaction of DNA through the formation of 5-hydroxy-methyl cytosine. Jumonji family of histone demethylases have a similar reaction mechanism (Chen et al., 2006). Both TeT and Jumonji enzymes use α-ketoglutarate as a co-factor, which is a key metabolite of the citric-acid cycle. Enzymes catalysing the formation of α-ketoglutarate are different isoforms of isocitrate dehydrogenase (IDH). Some of the IDHs are mitochondrial, others are cytosolic, and they depend on NADP and NAD, respectively. SNPs of these enzymes are associated with TS (see later), while their mutations show frequent occurrence in tumors (gliomas, AML) (Dang et al., 2010). The gain-of-function mutant enzymes catalyse the formation of 2-hydroxyglutarate (2-HG), a potent inhibitor of demethylases. Altogether these data suggest a tight link between environmental factors and epigenetic modifications.

### Non-coding RNA(ncRNA)

Evidence from the Encyclopedia of DNA Elements (ENCODE) suggests that at least 80% of our genome is transcribed. The human genome encodes for less than 3% of protein-coding transcripts and consists primarily of the non-coding RNAs (ncRNAs), which was previously regarded as “junk” DNA (https://www.encodeproject.org/). Non-coding RNAs play a role in gene expression regulation (see below). For example they are implicated in the regulation of genes coding for enzymes catalyzing epigenetic modifications. Furthermore, non-coding RNAs are also involved in the regulation of chromosomal domains in tight interaction with epigenetic covalent modifications (e.g., X-chromosome inactivation).

An arbitrary threshold of 200 nucleotides of transcript length was drawn to classify two groups of ncRNAs into small or long ncRNAs. Small ncRNAs include microRNAs (miRNA), transfer RNAs (tRNA) and small nucleolar RNAs (snRNA). A microRNA (miRNA) is a small non-coding RNA molecule found in plants, animals and some viruses, which functions in transcriptional and post-transcriptional regulation of gene expression.

**Figure 3:**
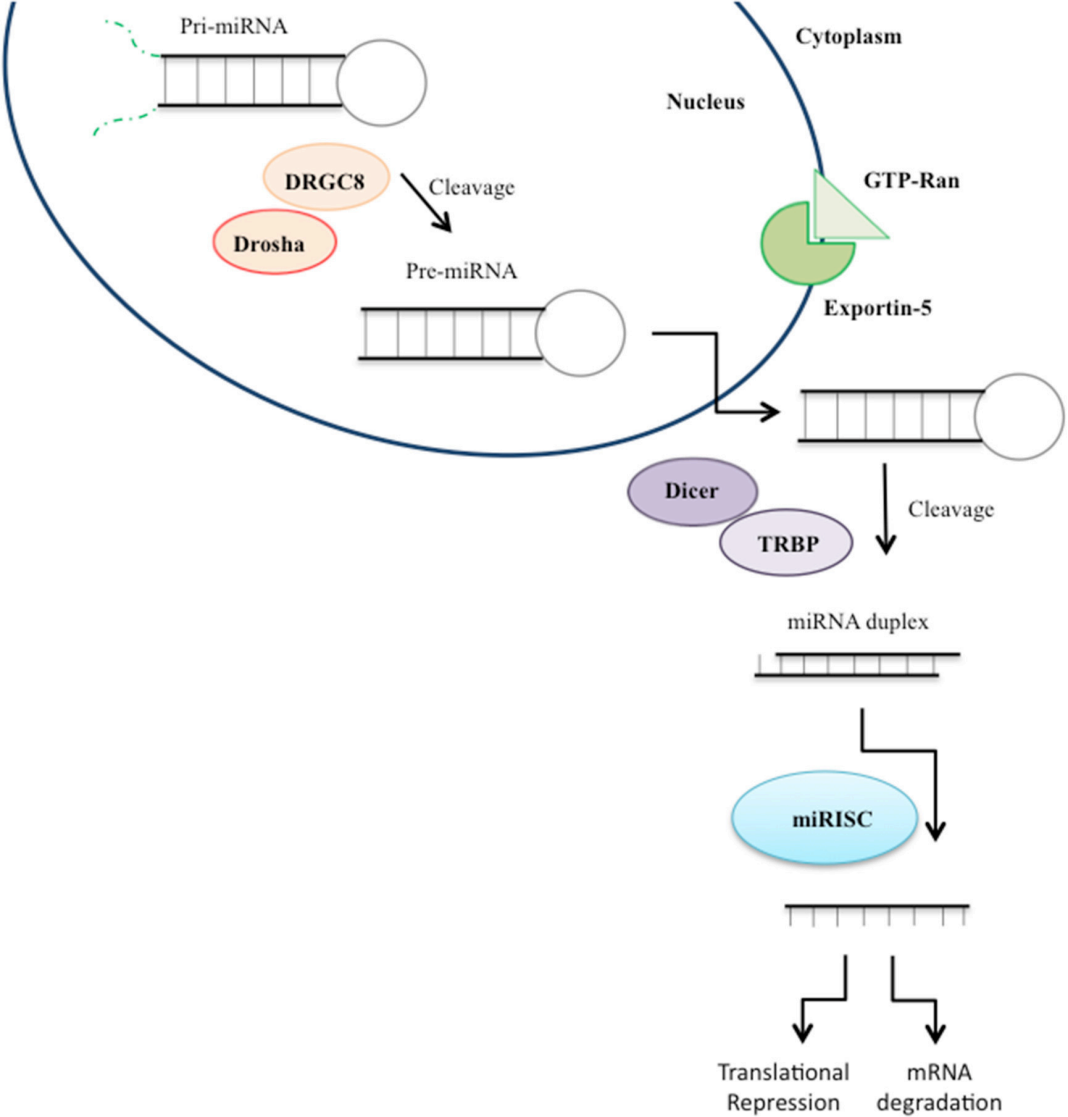
**miRNA biogenesis**. MicroRNA (miRNA) genes are transcribed as primary miRNAs (pri-miRNAs) by RNA polymerase II (Pol II) in the nucleus. The long pri-miRNAs are cleaved by Microprocessor, which includes DROSHA and DiGeorge syndrome critical region 8 (DGCR8), to produce precursor miRNAs (pre-miRNAs), which are then exported to the cytoplasm by Exportin 5 and further processed by the DICER/TRPB complex to produce an miRNA duplex. One strand of the mature miRNA (the guide strand) is loaded into the miRNA-induced silencing complex (RISC) mediating gene suppression by targeted mRNA degradation or translational repression.

The biogenesis of microRNA involves two different cleavage steps by protein complexes taking place in the nucleus and in the cytoplasm leading to the development of an ~22 nucleotide long mature single-stranded miRNA (Figure 3; Filipowicz et al., 2008). First, the gene coding for miRNAs is transcribed by RNA Polimerase II resulting in a long precursor pri-miRNA characterized by a hairpin or fold-back structure with an imperfectly base-paired stem and a terminal loop (Miyoshi et al., 2010). Then, the hairpin structure of the pri-miRNA is cleaved and the ~70 nucleotide long precursor called pre-miRNA is released (Miyoshi et al., 2010). Subsequently, the pre-miRNA is exported into the cytoplasm (Kim et al., 2009). Once in the cytoplasm, the pre-miRNA is processed by RNase III Dicer generating a ~22 nucleotide miRNA-miRNA*duplex. One strand of the duplex is loaded into a large multi-protein miRNA ribonucleoprotein complex (RISC complex), while the other strand, “passenger,” is degraded. Once incorporated into RISC, the miRNA guides the complex to its messenger RNA targets by base-pairing interactions. The binding to the miRNA target relies on the seed sequence, a 6–8 nucleotide domain located at the 5′ end of the miRNA. Based on the complementarity of the seed sequence and the target mRNA sequence located in the 3′UTRof the transcript, miRNAs down-regulate gene expression either through translational repression or mRNA degradation (Hutvágner and Zamore, 2002).

Longer RNAs include ribosomal RNAs (rRNA), natural antisense transcripts and other long non-coding RNAs (lncRNAs). Currently, about 2500 miRNAs and 50,000 lncRNAs have been annotated in the human genome, besides the ~19,000 protein coding genes. As opposed to miRNAs, only a few lncRNAs show evolutionary conservation of the primary sequence, but most of them show tissue and cell type-specific expression, indicating that their expression must be tightly controlled. LncRNAs exhibit a diversity of molecular functions: they can act as transcriptional activators or repressors, as scaffolds for protein-protein interactions or as molecular decoys.

Genetic polymorphisms can involve both ncRNA sequences throughout the genome, as well as sequences in their target genes, which can affect ncRNA-mediated regulation. Polymorphisms in ncRNA genes can influence both their level of expression and the ncRNA function, thus resulting in differential regulation of their target genes.

Genetic variation may affect miRNA-mediated regulation in different ways. Altered miRNA transcription can be a result of polymorphisms in (a) miRNA promoters, (b) splice sites of the host gene where miRNAs reside, since many miRNAs are intronic, or (c) of polycistronic miRNA clusters (Calore et al., 2015). Mutations in the sequence of the transcribed miRNA may change its binding affinity to the biogenesis enzyme complexes, which changes their processing and may lead to misregulation of target genes. IsomiRs are variants within the processed miRNA sequence, which can affect specificity to their target, on the other hand target messenger RNA 3′UTR variants may either destroy existing miRNA seed regions or create new recognition sites. The presence of a SNP in the 3′UTR can theoretically result in three alterations in miRNA related regulation: (a) it can partially or completely disrupt the miRNA binding site, thus resulting in higher expression of the target gene, or (b) more rarely a SNP could either enhance the binding of a miRNA to the 3′UTR region through improvement of the original recognition site or (c) it may create a novel binding site for another miRNA. The latter will only affect the expression of the target gene if it coincides with the expression of the new miRNA spatially and temporally.

Although lncRNAs possess a much lower degree of conservation than miRNAs, their genetic polymorphisms may still be functional, i.e., SNPs within the lncRNA loci can change their expression or influence their downstream target genes. Altering lncRNA architecture may influence its ability to interact with proteins or other RNAs. In recent years, genetic variations in lncRNAs were implicated in several human diseases.

## Epigenetics of neuropsychiatric disorders

Epimutations are epigenetic alterations, which have been linked to several diseases. These alterations can be classified as primary or secondary based on their origin (Oey and Whitelaw, 2014). Primary epimutations seem to be due to only environmental stress factors. It is often difficult to understand the pathomechanism of the resulting diseases or traits, which are generally less severe than in the case of secondary epimutations. Secondary epimutations are due to an initial genomic mutation. Most of these mutations target readers, writers and erasers of the epigenetic system and can lead to important changes of the global epigenetic profile (Lopez-Atalaya et al., 2014). In the following section we will describe some examples of epimutations leading to neuropsychiatric disorders (Plazas-Mayorca and Vrana, 2011).

### Rubinstein-Taybi syndrome

Rubinstein-Taybi syndrome has various clinical signs including moderate to severe learning difficulties (for a recent review see Lopez-Atalaya et al., 2014). The hereditary disease is autosomal dominant although very few documented cases of transmission exist. Most of the patients have *de novo* mutations. The development of the syndrome can be attributed to mutations in the CREB-binding protein (CBP) or more rarely in the highly homologous p300 protein encoding genes. Both proteins are transcriptional co-activators, have HAT activity and they bind the acetylated histones *via* their bromodomain. Thus, they provide platforms for other proteins (transcription factors and RNA PolII) necessary for transcription initiation.

### Gliomas

Gliomas (Vigneswaran et al., 2015) are tumors arising from glial cells in the central nervous system (CNS). During tumor progression patients experience psychiatric, cognitive, and neurologic symptoms (Boele et al., 2015). Primary low-grade gliomas are typically diagnosed in the 40 s, and after treatment, these slow-growing tumors have a tendency to reappear and progress in grade to become grade III gliomas or glioblastomas (grade IV). In contrast to the secondary high-grade glioblastomas, the primary high-grade glioblastomas are diagnosed later and have very poor prognosis. Although histologically identical, the primary and secondary glioblastomas have different molecular characteristics.

Both primary low-grade and secondary high-grade gliomas are characterized by IDH1/2 mutation (see above). Approximately 90% of the mutations occur in the *IDH1* gene and almost all of them are the R132H variant (Vigneswaran et al., 2015). As mentioned earlier, this enzyme variant catalyzes the formation of an oncometabolite (2-HG), which inhibits DNA and histone demethylation leading to general (secondary) alteration of the epigenetic profile (Cohen et al., 2013). Nevertheless, gliomas with mutated *IDH1/2* gene have better prognosis than the others (Andronesi et al., 2013).

### Rett syndrome

Rett syndrome is a disease affecting only girls (1:10,000–15,000) (Chahrour and Zoghbi, 2007; Katz et al., 2012) and it is characterized by early onset (18 months) and variable neurological symptoms. Severe mental retardation and motor impairment such as ataxia, apraxia, and tremor (Chahrour and Zoghbi, 2007) accompanied by seizures and gastrointestinal symptoms are frequently present (Katz et al., 2012). Rett syndrome is an example of a severe disorder due to the mutation of the MeCP2 gene encoding an epigenetic “reader” i.e., a regulatory protein recognizing an epigenetic mark (Amir et al., 1999). Therefore, although Rett syndrome is considered to be a typical epigenetic disease, no major epigenetic alterations can be observed in the patients. Loss-of-function mutations of the gene coding for the transcriptional repressor *MeCP2* are responsible for the development of the disease (Amir et al., 1999). MeCP2 is a member of the methyl-CpG binding domain (MBD) protein family. Upon binding of methylated DNA, MeCP2 recruits transcriptional repressor complexes and HDACs. Interestingly, MeCP2 has recently been found to bind and repress long genes implicated in neuronal differentiation and modulation of neuronal functions (Gabel et al., 2015).

### Autism spectrum disorder (ASD)

Rett syndrome is also considered to be a rare form of autism spectrum disorder (ASD) (Mbadiwe and Millis, 2013). ASD is clinically characterized by social communication deficits and repetitive behavior, which appears as early as 2 years of age and causes clinically significant impairment. ASD has high heritability rates suggesting a substantial genetic background. It has a polygenic origin with hundreds of susceptibility genes. Most of them are common variants with small effects, while some rare de novo variants with large effects also exist (Loke et al., 2015). Apart from *MeCP2, FMR1*, and *OXTR* also have profound effects and they were repeatedly reported in relation with ASD (Mbadiwe and Millis, 2013). Both of them are also linked to epigenetic alterations, which is clearly secondary in case of FMR1.

### Fragile X syndrome

Approximately half of the patients with Fragile X syndrome meet the criteria of autism and it is a relatively frequent cause of ASD (5% of all monogenic cases). The *FMR1* gene encodes FMRP, a polyribosome associated protein playing an important role in protein translation (Penagarikano et al., 2007). The absence of the protein leads to perturbed neuronal development and intellectual disability (Contractor et al., 2015). Fragile X syndrome is caused by a CGG trinucleotide expansion in the regulatory region of the *FMR1* gene located on the chromosome X. This repeat expansion (>200) leads to the attraction of DNA methylation and the loss of expression of the gene (Oberlé et al., 1991; Penagarikano et al., 2007).

The oxytocin receptor (OXTR) gene is also a candidate gene for ASD (Loke et al., 2015). The neurotransmitter and hormone oxytocin was found to play a role in anxiety, aggressive behavior, and other neural functions. Several observations indicate that OXTR plays a role in the development of ASD. For instance, four SNPs in the gene were suggested to be associated with ASD. Furthermore, several studies reported higher DNA methylation level in patients than in controls in the promoter region of the gene (Gregory et al., 2009; Jack et al., 2012; Ziegler et al., 2015). Very important methylation increase (20–40%) was observed both in temporal cortex and peripheral blood. This temporal hypermethylation was also correlated with lower OXTR mRNA levels in autists (Gregory et al., 2009). Thus, it is not surprising that OXTR methylation has been associated with anxiety disorder and other traits characterizing ASD.

A recent genome-wide analysis of DNA methylation studied a sample of 50 monozygotic (MZ) twin pairs including twins discordant, as well as concordant for ASD. Within-twin and between-group analyses identified a number of differentially methylated regions associated with ASD. In addition, the authors reported significant correlations between DNA methylation and quantitatively measured autistic trait scores across the cohort implicating a role for altered DNA methylation in autism (Wong et al., 2014).

Finally, strong evidence shows that ASD has primary epigenetic origin, as well (Tordjman et al., 2014). Children with *in utero* exposure to the HDAC inhibitor valproic acid (an anticonvulsive and mood stabilizer drug) were found in several studies (Moore et al., 2000; Bromley et al., 2013; Christensen et al., 2013) to have a significantly increased risk to develop autism relative to those who were not treated. Other environmental factors during pregnancy can be considered as risk factors for ASD, such as viral infection (e.g., rubella) (Ornoy et al., 2015) and the dietary folic acid supplementation, which is regarded as controversial (Yang et al., 1989; Mbadiwe and Millis, 2013). Finally, several studies indicate that prenatal maternal stress is also a risk factor for developing ASD (Kinney et al., 2008 and references therein).

A considerable number of studies indicate that early life adversities (ELA) (e.g., childhood abuse or even prenatal and/or maternal stress) are severe risk factors for the development of psychiatric disorders such as major depression, suicidal behavior, etc. (Hoffmann and Spengler, 2014; Palma-Gudiel et al., 2015; Cattaneo and Riva, 2016). This seems to be due to a difficulty in coping with stress in these patients. During stress reactions the hypothalamus-pituitary-adrenal axis (HPA) is activated and glucocorticoids (cortisol in humans and corticosterone) are released, which in turn activate the pathways regulated by glucocorticoid and mineralocorticoid receptors. The axis is inhibited by the feedback activation of the glucocorticoid receptor (GR) in the hippocampus (Palma-Gudiel et al., 2015; Cattaneo and Riva, 2016).

The molecular mechanisms of the development of ELA-related alterations have been deciphered in a rat model system (Weaver et al., 2004). Weaver and colleagues have compared pups from “good” nursing and “bad” nursing females, two maternal behaviors generally occurring in rats (Liu et al., 1997; Caldji et al., 1998). Offsprings of “good moms” were less fearful and had a lower stress response in their adulthood than those of “bad moms.” Weaver and colleagues observed higher DNA methylation levels in the hippocampus from early childhood (after postnatal day 1) until at least 3 months of age in the GR promoter in the offsprings of “bad moms” relative to those of “good moms.” This difference concentrated at a certain region of the promoter and more precisely at a single CpG, located at the binding site of transcription factor NGFI-A regulating GR expression. They also observed the decreased binding of NGFI-A and hypoacetylation of histones in the methylated region of the promoter. These findings were accompanied by lower GR expression. Interestingly, but not surprisingly for an epigenetic mark, both the molecular and the behavioral phenotypes were reversible. Treatment with Trichostatin A, an HDAC inhibitor, reversed histone hypoacetylation, increased NGFI-A binding, GR expression, and decreased DNA methylation to some extent. Similarly, changing the environment had a similar effect as shown by cross-fostering, demonstrating that this phenotype is not determined by the genetic background and should be considered as having a primary epigenetic origin.

Based on these observations, several studies in animal models confirmed these findings (McGowan et al., 2011). In human cohorts a very small, but systematic methylation increase was reported from the same region of the GR promoter in individuals undergoing stressful events (Palma-Gudiel et al., 2015).

Non-coding RNAs are also associated with a wide range of neurodevelopmental, neurodegenerative, and psychiatric diseases both in humans and in animal models (Lin et al., 2011; Johnson, 2012; Talkowski et al., 2012; Ziats and Rennert, 2012; Nishimoto et al., 2013; Petazzi et al., 2013; Barry et al., 2014). As a recent example, genetic variants of the long non-coding RNA MIAT were found to contribute to the risk of paranoid schizophrenia in a Han Chinese population (Rao et al., 2015). The authors performed a two-stage association analysis on 8 tagging SNPs covering the whole *MIAT* locus in two independent Han Chinese schizophrenia case–control cohorts. The discovery sample with over 1000 cases and 1000 controls yielded a significant increase of the minor T-allele of rs1894720 in patients and this association was confirmed in the replication cohort of a similar size.

MicroRNAs are also implicated in several neuropsychiatric disorders, such as schizophrenia and autism (Beveridge and Cairns, 2012; Mellios and Sur, 2012), neurodegenerative disorders like Alzheimer's and Parkinson's disease (Salta and De Strooper, 2012; Abe and Bonini, 2013; Tan et al., 2013), but also in other neurodevelopmental disorders such as Fragile X syndrome and Rett syndrome (Urdinguio et al., 2010; Wu et al., 2010; Im and Kenny, 2012; Sellier et al., 2013).

### Epigenetics of tourette syndrome

As described earlier, the studies investigating TS mainly focused on the genetic background of the disease. Only few studies have been performed to date to investigate the role of epigenetic factors and non-coding RNA in the development of TS. One of these identified a nucleotide variant (var321) in the 3′ UTR of the *SLITRK1 g*ene leading to its stronger repression by miR-189. This variant has been investigated in several studies in Tourette patients and reported to be rare (Abelson et al., 2005). However, the role of this variant in TS pathogenesis is questionable due to its very low frequency (Keen-Kim et al., 2006). The unique study reported to date on the role of microRNAs in Tourette Syndrome profiled the expression of 754 miRNAs in the sera of six TS patients and three unaffected controls (Rizzo et al., 2015). The study found that miR-429, which is involved in midbrain and hindbrain differentiation and synaptic transmission was significantly underexpressed in TS patients. Measurement of circulating miR-429 may in the future be useful as a molecular biomarker to aid TS diagnosis.

Two association studies on DNA methylation related to TS have been conducted so far. The first showed no methylation alteration in TS patients relative to controls in a region on chromosome 8 in KCNK9 and TRAPPC9 genes (Sánchez Delgado et al., 2014). The regions were identified recently by genome-wide screens and by mapping mutations in single families. The other study was the first Epigenome-Wide Association Study (EWAS) investigating DNA methylation differences between hundreds of controls and patients from the Netherlands Twin Registry with tic phenotype (Zilhão et al., 2015). Very small methylation differences were observed, however, an enrichment of differentially methylated neural genes previously linked to neuropsychiatric disorders or with brain specific function was found among the top hits.

Finally, a recent promising study investigated GWAS results on gene sets. The association of TS and a gene set related to carbohydrate metabolism and more particularly a group of 33 genes involved in “astrocyte-neuron metabolic coupling,” glycolysis and Krebs-cycle was demonstrated (de Leeuw et al., 2015). This is interesting because the genes identified include IDH2 (see above) and Malic enzyme 1, which is regulated by glucose level. Since these TS associated metabolic genes are known to play a role in epigenetic modifications, this suggeststhat the disorder is potentially characterized by altered neural epigenetic patterns.

### Outlook

In the present review, we have shown that TS is a neuropsychiatric disorder with significant heritability. However, while very few rare genetic variants with large effects were described, it is plausible to assume that hundreds or even more frequent variants with small effects underlie the genetic susceptibility of the disorder. Furthermore, a considerable number of observations indicate that environmental factors also play a crucial role in the development of TS. As introduced above, environmental factors act *via* epigenetic modifications, including heritable covalent modifications of the chromatin and regulatory non-coding RNAs. In order to better understand the pathomechanism of TS, we propose here that more studies should be performed focusing on the role of epigenetics.

What questions should be asked? We think that since environmental (risk) factors are implicated in TS, these should be studied with particular interest. For instance, discordant monozygotic twins are very good candidates for finding epigenetic modifications implicated in the development of the disease, since they are genetically identical. Similarly, patients with known prenatal or perinatal antecedents probably also have important epigenetic grounding in the development of TS. We also consider that patients who developed TS due to streptococcus infection should also show epigenetic alteration relative to controls. Finally, since the disease is characterized by waxing and waning, kinetic analysis of epigenetic changes might also reflect important aspects of TS.

Animal models of TS or tic phenotype should also be studied for epigenetic alterations. While studies on human cohorts might be more descriptive, analyses of animal models could explain more directly the underlying molecular mechanisms.

How should these questions be addressed? Currently several techniques are available to study epigenetics. Genome-wide analyses are much more informative than investigations of single targets. These genome-wide approaches have recently become much less expensive and therefore affordable for most of the laboratories or consortia. Expression level and GWAS analyses can be performed on ncRNA, while chromatin immunoprecipitation (ChIP) followed by next generation sequencing (NGS) is useful for the investigation of histone modifications (Furey, 2012). Along with the ongoing technological advances in NGS, identifying genetic variation affecting ncRNA function associated with neuropsychiatric disease is likely to grow rapidly.

Finally, several genome-wide techniques exist for the study of DNA hydroxymethylation and DNA methylation. We propose to study DNA methylation rather than histone modifications, because it is more stable, than the latter one, although not as static as initially thought (Yamagata et al., 2012b). Furthermore, ChIP-like antibody-based techniques are much less quantitative than the chemical transformation (bisulfite conversion-based) techniques developed for DNA methylation. Therefore either array-based hybridization approaches after bisulfite conversion [Illumina 450 k (Bibikova et al., 2011) or the recent 850 k bead chips] or bisulfite conversion coupled to next generation sequencing could yield useful information (Kulis et al., 2012).

What cautions should be taken? First, we consider that the most important issues are sample size and tissue of origin. TS is a psychiatric disease mainly due to alterations in neuronal and potentially in glial cells. These cells in humans are rarely available for research, while blood cells and buccal or nasopharingeal epithelial cells have somewhat different epigenetic signatures. Although some of these cells can have similar epigenetic profiles in some genomic regions to the most important brain regions in TS (e.g., striatum), the results should always be interpreted cautiously (Hannon et al., 2015). In order to avoid such problems, brain samples should be investigated when possible, however that has obvious limitations in humans. Alternatively, as mentioned before discordant monozygotic twins can also be studied, finally, patients with TS presumably due to PANDAS origin are good candidates for blood sample analysis. It is hard to determine the ideal sample size. However, it is clear that already small sample sizes can be informative if repeated experiments give similar results and the samples are well-selected.

Second, new approaches available and proposed lead to the generation of “big data.” Their correct analysis is necessary and requires the intensive collaboration of the biomedical and bioinformatitian scientists with profound knowledge of statistics.

Finally, when interpreting data, attention and caution should be exercised. Although biologically meaningful cutoffs for methylation differences between patients and controls is hard to determine, the value of very small but statistically significant changes is questionable. Furthermore, statistically significant hits showing association with the phenotype does not neccessarily mean causality. From a single descriptive experiment causality cannot be concluded, but further experiments should be performed.

In conclusion, we consider that introducing epigenetic studies in TS research has great potential. These investigations based on the previous results, the animal models and the twin and patients registries already existing will certainly identify new molecular mechanisms and hits playing an important role in the development of the disease. The discovery of the molecular details of ncRNA and epigenetic modification mediated regulation of gene expression, proteins and pathways is likely to provide novel insights into the pathogenesis of neuropsychiatric disease including Tourette Syndrome. This will also open new avenues for genetic diagnosis, as well as targeted and personalized therapeutic approaches. These studies will also strengthen the importance of some already known and suspected hits and altogether this will lead to the better understanding of TS and the opening of new avenues for the development of treatments.

## Author contributions

LP, BV, TA, and CB were all involved in building up the concept of the paper, literature research, and writing of the manuscript.

### Conflict of interest statement

The authors declare that the research was conducted in the absence of any commercial or financial relationships that could be construed as a potential conflict of interest.
